# NEK2 Promotes Aerobic Glycolysis in Multiple Myeloma Through Regulating Splicing of Pyruvate Kinase

**DOI:** 10.1186/s13045-017-0392-4

**Published:** 2017-01-13

**Authors:** Zhimin Gu, Jiliang Xia, Hongwei Xu, Ivana Frech, Guido Tricot, Fenghuang Zhan

**Affiliations:** 1 Department of Medicine, Division of Hematology, Oncology and Blood and Marrow Transplantation and Holden Comprehensive Cancer Center, University of Iowa, 585 Newton Rd, 52242 Iowa City, IA USA; 2 Institute of Cancer Research, School of Basic Medical Sciences, Southern Medical University, Guangzhou, China

**Keywords:** NEK2, Pyruvate kinase, Multiple myeloma, Alternative splicing

## Abstract

**Background:**

Aerobic glycolysis, a hallmark of cancer, is characterized by increased metabolism of glucose and production of lactate in normaxia. Recently, pyruvate kinase M2 (PKM2) has been identified as a key player for regulating aerobic glycolysis and promoting tumor cell proliferation and survival.

**Methods:**

Tandem affinity purification followed up by mass spectrometry (TAP-MS) and co-immunoprecipitation (Co-IP) were used to study the interaction between NIMA (never in mitosis gene A)-related kinase 2 (NEK2) and heterogeneous nuclear ribonucleoproteins (hnRNP) A1/2. RNA immunoprecipitation (RIP) was performed to identify NEK2 binding to PKM pre-mRNA sequence. Chromatin-immunoprecipitation (ChIP)-PCR was performed to analyze a transcriptional regulation of NEK2 by c-Myc. Western blot and real-time PCR were executed to analyze the regulation of PKM2 by NEK2.

**Results:**

NEK2 regulates the alternative splicing of PKM immature RNA in multiple myeloma cells by interacting with hnRNPA1/2. RIP shows that NEK2 binds to the intronic sequence flanking exon 9 of PKM pre-mRNA. Knockdown of NEK2 decreases the ratio of PKM2/PKM1 and also other aerobic glycolysis genes including GLUT4, HK2, ENO1, LDHA, and MCT4. Myeloma patients with high expression of NEK2 and PKM2 have lower event-free survival and overall survival. Our data indicate that NEK2 is transcriptionally regulated by c-Myc in myeloma cells. Ectopic expression of NEK2 partially rescues growth inhibition and cell death induced by silenced c-Myc.

**Conclusions:**

Our studies demonstrate that NEK2 promotes aerobic glycolysis through regulating splicing of PKM and increasing the PKM2/PKM1 ratio in myeloma cells which contributes to its oncogenic activity.

## Background

In the 1920s, Dr. Otto Heinrich Warburg observed that cancer cells uptake more glucose compared with normal tissues and metabolize glucose via glycolysis, a low efficient pathway for generating ATP, rather than mitochondrial oxidative phosphorylation, regardless of oxygen availability [1–3]. This process is now known as “Warburg effect” or aerobic glycolysis. In the past decades, researches confirmed that aerobic glycolysis is the hallmark of cancer cells and important for their proliferation and survival [4–9]. In addition to generating energy, aerobic glycolysis is involved in the biosynthesis of cancer cells. The intermediate of glycolysis is used as a carbon source for the generation of nucleic acids, phospholipids, fatty acids, cholesterol, and porphyrins [1, 6, 8]. Aerobic glycolysis also affects tumor microenvironment. In cancer cells, glucose is metabolized to lactate through glycolysis, and then the lactate is released outside the cells by monocarboxylate transporters. The release of lactate results in environmental acidosis, which protects cancer cells against attack from the immune system [1, 6, 8]. Additionally, aerobic glycolysis was found to affect the cells signaling of tumor cells through maintaining the appropriate balance of reactive oxygen species (ROS) and histone acetylation [1, 6, 8]. The inhibition of Warburg effect deprives the generation of ATP, decreasing cancer cells growth and proliferation [10, 11]. Thus, Warburg effect has received substantial attention as a novel therapeutic target in cancers including lung cancer [12, 13], leukemia [14], breast cancer [15–18], pancreatic cancer [19, 20], colorectal cancer [21, 22], bladder cancer [23], and multiple myeloma [24, 25]. In multiple myeloma, dichloroacetate, which is an inhibitor of aerobic glycolysis, has been reported to increase myeloma cell sensitivity to bortezomib [24]. Additionally, inhibition of aerobic glycolysis was found to contribute to melphalan treatment in myeloma [25]. Pyruvate kinase (PK) is one of the key regulators of the Warburg effect that convert phosphoenolpyruvate (PEP) to pyruvate and generate one molecular of ATP [26, 27]. PK family consists of four isoforms: liver‑type PK (PKL), red blood cell PK (PKR), and PK muscle isozyme M1 and M2 (PKM1 and PKM2, respectively) [27, 28]. PKM1 and PKM2, produced by an alternative splicing of the primary RNA transcript of the PKM gene, play important roles on Warburg effect. PKM1 is constitutively activated and expressed in terminally differentiated tissues to promote oxidative phosphorylation, whereas PKM2 is highly expressed in embryonic and cancer cells, which is an allosteric isoform and exhibits a dimer with low affinity for PEP. Tetramer PKM2 exhibits highly catalytic activity leading to ATP synthesis and catabolic metabolism. In contrast, dimeric PKM2, which is the low active state of PKM2, accelerates glycolytic intermediates to enter the glycolysis, such as glycerol synthesis and the pentose phosphate pathway [26–30]. Increased PKM2/PKM1 ratio has been reported in multiple cancers and has been closely associated with shorter overall survival in cancer patients [31–36]. Understanding the regulation of PKM pre-mRNA alternative splicing is of great importance for developing cancer therapy. The splicing factors of heterogeneous nuclear ribonucleoproteins (hnRNP) A1/2 and polypyrimidine-tract binding (PTB) protein, which mediate c-Myc enhanced PKM2/PKM1, drive alternate splicing of PKM pre-mRNA by selectively inclusion of exon 10 and the exclusion of exon 9 [37–39].

Never in mitosis (NIMA)-related kinase 2 (NEK2) is a serine/threonine kinase that promotes centrosome splitting and ensures correct chromosome segregation during the G2/M phase of the cell cycle [40]. Former studies from our group and others have indicated that NEK2 promotes tumor cell proliferation, tumor progression, and drug resistance. High expression of NEK2 is associated with poor survival in various cancers [41–44]. Naro et al. reported that NEK2 is localized at the splicing speckles and phosphorylates the oncogenic splicing factor SRSF1 [45]. We recently performed a tandem affinity purification followed up by mass spectrometry (TAP-MS) analysis and identified that NEK2 binds to hnRNPA proteins in myeloma cells. Therefore, we hypothesize that NEK2 regulates alternative splicing of PKM2/PKM1 through interacting with hnRNPA proteins, leading to modulation of aerobic glycolysis in myeloma cells. In this study, we determine whether NEK2 increases PKM splicing and PKM2 expression resulting in high aerobic glycolysis in myeloma cells using engineered isogenic myeloma cell lines with over or lower expression of NEK2. We also explore whether NEK2 is a direct target of the transcription factor c-Myc. In summary, our studies show the first evidence that NEK2 plays a functional role in aerobic glycolysis and provide mechanistic insights how NEK2 promotes aerobic glycolysis in myeloma.

## Methods

### Gene expression profiling

The data of gene expression profile (GEP), which were derived from NIH Gene Expression Omnibus (http://www.ncbi.nlm.nih.gov/geo/), include 22 healthy subjects (accession number GSE5900), 44 monoclonal gammopathy of undetermined significance (MGUS) patients (accession number GSE5900), 305 low-risk, and 46 high-risk myeloma patients (accession number GSE2658). Affymetrix U133Plus2.0 microarrays were used to analyze these samples as previously described [46]. Signal intensities were preprocessed and normalized by GCOS1.1 software. The expression and relationship between NEK2, c-Myc, PKM2, and aerobic glycolysis relative genes were investigated in these samples.

### Cell culture

Human myeloma cell lines, ARP1, OPM2, and the B cell line P493-6 (a gift from Dr. Thomas-Tikhonenko, University of Pennsylvania) were cultured at 37 °C and 5% CO_2_ in RPMI 1640 (Gibco, Grand Island, NY) supplemented with 10% heat inactivated fetal calf serum (Gibco, Grand Island, NY) and 1% penicillin and streptomycin (Gibco, Grand Island, NY).

### Western blotting

Briefly, total proteins from myeloma cells were extracted using Mammalian Cell Extraction Kit (K269–500, Biovision, Milpitas, CA). Protein samples (20 μg/sample) were separated using SDS-PAGE and transferred into the nitrocellulose membrane via Bio-Rad Mini-Protean electrotransfer system. The membranes were blocked with 5% non-fat dry milk in TRIS buffered saline (TBS) containing 0.05% Tween-20 (TBST) prior to incubation overnight at 4 °C with primary antibody including NEK2 (Santa Cruz, USA), FLAG (Sigma, USA), HA (C29F4), PKM2 (D78A4), c-Myc (D84C12), hnRNPA1 (D21H11), hnRNPA2 (A2A), and β-actin (D6A8) (Cell Signaling, USA). Respective HRP-conjugated secondary antibodies were added and protein signals were developed with the use of the HRP substrate luminol reagent (Millipore, CA). The developed images were obtained and analyzed using ChemiDoc^TM^ XRS+System (Bio-Rad, USA).

### Co-immunoprecipitation

Co-immunoprecipitation (Co-IP) was performed as previously describe [47] with some modifications. Briefly, total proteins from NEK2 overexpressing ARP1 cells were extracted with IP lysis buffer (Thermo Scientific, USA). HA antibodies (C29F4, Cell Signaling, USA) or control immunoglobulin (IgG) (Cell Signaling, USA) were added and incubated with cell lysate overnight at 4 °C. Then followed by protein A Dynabeads (Invitrogen, USA) incubation for 2 h at 4 °C. The beads were washed three times with TBST (Sigma, USA). The pulled-down proteins were extracted and examined by Western blotting as described above.

### RNA immunoprecipitation

RNA immunoprecipitation (RIP) was carried out as previously described [48] with some modifications. Briefly, NEK2 overexpressing ARP1 cells were cross-linked with 1% formaldehyde (Covaris, USA) for 5 min at room temperature and then followed by Covaris quenching buffer (Covaris, USA) incubation for 5 min to stop the cross-link. Cells were lysed by Covaris lysis buffer (Covaris, USA) containing protease inhibitor cocktail (Covaris, USA) and RNase inhibitor (Invitrogen, USA). Nuclear pellets were collected and lysed through sonication. Nuclear lysates were incubated with HA antibody or control IgG conjugated protein A Dynabeads (Invitrogen, USA) overnight at 4 °C followed by stringent washing of bead pellets with final resuspension in TRIzol (Invitrogen, USA). Co-precipitated RNAs were isolated and RT-PCR was performed to determine the sequence of EI9. EI9 forward and reverse primer sequences, respectively, 5′-TGCATGCTTCCACAGGCATC-3′; EI9 reverse primer 5′-TGGGCTAACTTGTGAGAGGC-3′.

### Immunofluorescence

Cells (1 × 10^5^) were spun down on glass slides and then fixed with 4% paraformaldehyde solution (Affymetrix, USA) for 15 min at room temperature. NEK2, hnRNPA1, and hnRNPA2 antibodies were diluted in TBS buffer with 0.1%, Triton 100, and 1% BSA. These antibodies were dripped on glass slides and incubated overnight at 4 °C. After 3 washes with TBST, secondary antibodies coupled to Alexa-Fluor® 488 goat anti-rabbit IgG(H+L) (Invitrogen, USA) or Alexa-Fluor® 594 goat anti-mouse IgG(H+L) (Invitrogen, USA) were added and incubated for 1 h at room temperature. Nuclei were labeled with DAPI (Vector Laboratories, CA). Fluorescence was observed under a fluorescence microscope.

### Quantitative real-PCR

Total RNA was extracted using RNeasy RNA isolation kit (Qiagen, USA) according to the manufacturer’s instructions. After digestion with RNase-free DNase (Roche, USA), 200 μg of total RNA was retrotranscribed using the 5×iScript^TM^ RT Supermix (Bio-Rad, USA). PCR primers were purchased from Integrated DNA Technologies (Coralville, IA). Real-time quantitative PCRs (qPCR) were performed using iTaq^TM^ Universal SYBR® Green Supermix (Bio-Rad, USA). Fold changes were calculated using the ΔΔCt method and glyceraldehyde 3-phosphate dehydrogenase (GAPDH) mRNA as reference. Primer sequences are listed in Table 1.

**Table 1:** Primer sequences for real-time PCR

Gene	Forward primers	Reverse primers
PKM1	5′-CGAGCCTCAAGTCACTCCAC-3′	5′-GTGAGCAGACCTGCCAGACT-3′
PKM2	5′-ATTATTTGAGGAACTCCGCCGCCT-3′	5′-ATTCCGGGTCACAGCAATGATGG-3′
GLUT4	5′-GCCATGAGCTACGTCTCCATT-3′	5′-GGCCACGATGAACCAAGGAA-3′
ENO1	5′-TGCGTCCACTGGCATCTAC-3′	5′-CAGAGCAGGCGCAATAGTTTTA-3′
LDHA	5′-ATCTTGACCTACGTGGCTTGGA-3′	5′-CCATACAGGCACACTGGAATCTC-3′
MCT4	5′-TCACGGGTTTCTCCTACGC-3′	5′-GCCAAAGCGGTTCACACAC-3′
HK2	5′-CAAAGTGACAGTGGGTGTGG-3′	5′-GCCAGGTCCTTCACTGTCTC-3′
NEK2	5′-CGGAAGTTCCTGTCTCTGGCA-3′	5′-TTCAGGTCCTTGCACTTGGACT-3′
GAPDH	5′-CTCTCTGCTCCTCCTGTTCGAC-3′	5′-TGAGCGATGTGGCTCGGCT-3′
NEK2 ChIP	5′-GTTCCAGTACCCTGAACCTGGGTG-3′	5′-GCCCACCCGGGAGTCTGTATTTC-3′
Negative ChIP	5′-CAGACCCGCTAAAGCTCAG-3′	5′-GGCTGGTTTTTCGGACCTAC-3′

*PKM1* pyruvate kinase isozymes M1, *PKM2* pyruvate kinase isozymes M2, *GLUT4* glucose transporter type 4, *ENO1* enolase 1, *LDHA* lactate dehydrogenase A, *MCT4* monocarboxylate transporter 4, *HK2* hexokinase 2, *NEK2* NIMA-related kinase 2, *GAPDH* glyceraldehyde 3-phosphate dehydrogenase

### Glucose uptake and lactate production assay

Glucose uptake was detected by a glucose uptake assay kit (Biovision, CA). Myeloma cells or P493-6 cells were plated into a 96-well plate. Cells were washed 3 times with PBS and then starved by preincubating with 100 μL Krebs Ringer Phosphate Hepes (KRPH) buffer for 40 min. Cells were stimulated with or without insulin (1 μM) for 20 min to activate glucose transporter, and 10 μL of 10 mM 2-deoxyglucose (2-DG) was added and incubated for 20 min. Cells were lysed with 90 μL of extraction buffer and then frozen/thawed once and heated at 85 °C for 40 min. The cell lysate was neutralized by adding 10 μL of neutralization buffer. The glucose uptake was measured by the cellular fluorescence (Ex/Em = 535/587 nm) in a microplate reader (BioTake, USA).

Lactate production was detected by a lactate assay kit (Biovision, CA). Myeloma cells or P493-6 cells were cultured in fresh phenol free RPMI1640 medium, and the culture medium was collected at the indicated times. Mix the culture medium with lactate assay buffer to 50 μL/well in a 96-well plate. Then 50 μL reaction buffer was added to every well and incubated for 30 min at room temperature. The lactate production was measured by the absorbance (570 nm) in a microplate reader.

### Flow cytometry

FITC-conjugated annexin V (eBioscience, USA) was used to label apoptosis cells. Dead cells were labeled by propidium iodide (PI) (eBioscience, USA). Staining experiment was performed according to the product instructions. Cells were then analyzed for apoptosis by flow cytometry (FACS) using the Cell Quest software. The results were analyzed using FlowJo software.

### Statistical analysis

All data were analyzed using two-tailed Student’s *t* test and expressed as mean ± SD between two groups. The difference of gene expression in multiple groups was analyzed by one-way ANOVA. A *p* value of 5% (*p < 0.05) was considered significant. Event-free (ES) and overall survivals (OS) were presented by the Kaplan-Meier curves, and the log-rank test was used to determine significance between gene expression levels with patient outcome. Significance was set at *p* < 0.05.

## Results

### NEK2 interacts with hnRNPA1/2 in myeloma cells

**Fig. 1:**
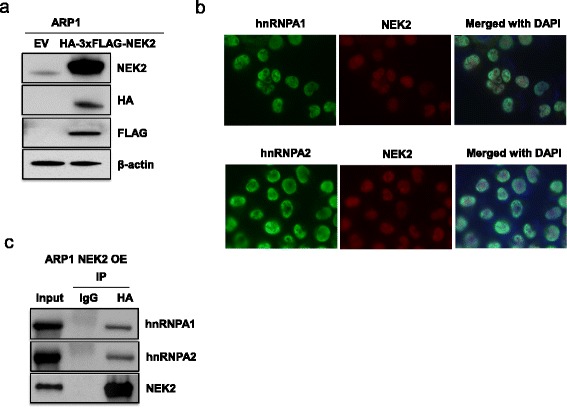
NEK2 interacts with hnRNPA1/2 proteins. **a** Western blots confirm that NEK2 cDNA conjugated with tags HA-3xFLAG are transfected into the myeloma cell line ARP1. **b** Immunofluorescence analysis of ARP1 cells stained with NEK2 antibody (*red*), hnRNPA1/2 (*green*), and DAPI (*blue*). **c** HA antibody was used to pull down NEK2, and its interacting proteins were analyzed by Western blotting. The lysates before IP were used as a positive control and IgG pulled down proteins as a negative control

NEK2 has been identified as an oncogenic protein which promotes tumorigenesis, tumor progression, and drug resistance. In this study, a TAP-MS analysis was performed to identify NEK2 interacting proteins in myeloma cells. To reduce nonspecific binding, plasmids containing human NEK2-cDNA tagged with HA and 3xFLAGS were transfected into a human myeloma cell line ARP1 by lentiviral delivery. Western blotting results confirmed that NEK2 was overexpressed in myeloma cells (Fig. 1a). NEK2 and its binding proteins were pulled down using sequential HA and Flag antibodies immunoprecipitation, and proteins bound to NEK2 were identified by mass spectrometry. The TAP-MS analysis showed that NEK2 binds at least to 67 proteins (data not shown), and the major functional category is the splicing factor family. We were particularly interested in hnRNPA2 because it is the key regulator of PKM pre-mRNA alternative splicing. It is known that hnRNPA2 forms a heterodimer with hnRNPA1 to play its biological function [39]. To confirm the interaction between NEK2 and hnRNPA1/2, immunofluorescence and Co-IP experiments were performed. Immunofluorescence images showed that NEK2 is co-localized with hnRNPA1/2 in the nucleus (Fig. 1b). As NEK2 protein was tagged with HA, we used HA antibody to immunoprecipitate NEK2, then detecting NEK2, hnRNPA1, and hnRNPA2 by Western blotting. As shown in Fig. 1c, NEK2, hnRNPA1, and hnRNPA2 were detected in HA immunoprecipitated proteins but not in IgG control. These results indicate that NEK2 binds to hnRNPA1 and hnRNPA2 proteins. This is consistent with a recent study that NEK2 interacts and activates several splicing factors as a novel splicing factor kinase [45]. Based on these data, we hypothesize that NEK2 may be involved in hnRNPA1/2 mediated pre-mRNA alternative splicing of PKM gene.

### NEK2 regulates the PKM2/PKM1 complex in myeloma cells

**Fig. 2:**
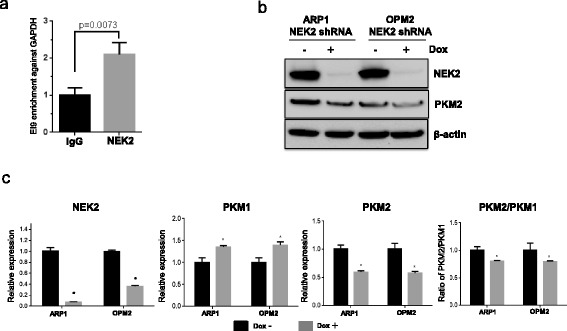
High NEK2 increases the ratio of PKM2/PKM1. **a** RNA immunoprecipitation using anti-HA antibody to pull down NEK2 binding RNA in ARP1 NEK2-HA OE cells. Real-time PCR was performed to test the enrichment of intronic sequence flanking exon 9 of PKM pre-mRNA. All values were normalized by genomic GAPDH, and IgG was used as negative control, *p < 0.05. **b** Western blots were performed to test the levels of NEK2 and PKM2 in NEK2-shRNA ARP1 and OPM2 MM cells. **c** Real-time PCR analyses of the ratio of PKM2/PKM1 in NEK2 knocked down ARP1 and OPM2 MM cells. Results of real-time PCR were normalized against GAPDH and presented means ± SD of triplicate determinations from an experiment representative of three, *p < 0.05

The hnRNPA1/2 complex binds to the intronic sequences flanking exon 9 of PKM pre-mRNA leading to exon 9 exclusion and exon 10 inclusion [37, 38]. In cancer or embryonic cells, increased hnRNPA1/2 proteins by c-Myc or others promotes exon 10 splicing and inclusion resulting in generation of pyruvate kinase isozyme type M2 (PKM2) [39]. We have confirmed that NEK2 binds with hnRNPA1/2 in myeloma cells described above, we then determined whether high NEK2 enhances its binding to the intronic sequences flanking exon 9 of PKM pre-mRNA. The RIP using HA-tag antibodies was performed to pull down NEK2 binding RNA sequences, and real-time PCR revealed that the intronic sequences flanking exon 9 of PKM pre-mRNA was significantly enriched in the NEK2 binding RNA compared with the IgG control (Fig. 2a). We further examined whether NEK2 regulates alternative splicing of PKM pre-mRNA in NEK2 silencing myeloma cells. NEK2 expression and PKM2 expression showed a decrease after addition of doxycycline by Western blotting in ARP1 and OPM2 myeloma cells (Fig. 2b). The expression of PKM1 and PKM2 was measured by real-time PCR in myeloma cells with or without knockdown of NEK2. Clearly, inhibition of NEK2 upregulated PKM1 expression but downregulated PKM2 (Fig. 2c). The ratio of PKM2/PKM1 was significantly decreased in myeloma NEK2-silenced cells (Fig. 2c). Since NEK2 is also localized in the nucleus, it is possible that NEK2 directly binds to the PKM pre-mRNA and regulates its splicing. If this is the case, we can prove it by pulling down RNA sequences using anti-NEK2 antibodies and determine if PKM pre-mRNA can be detected by PCR in future studies.

### NEK2 promotes aerobic glycolysis in myeloma cells

**Fig. 3:**
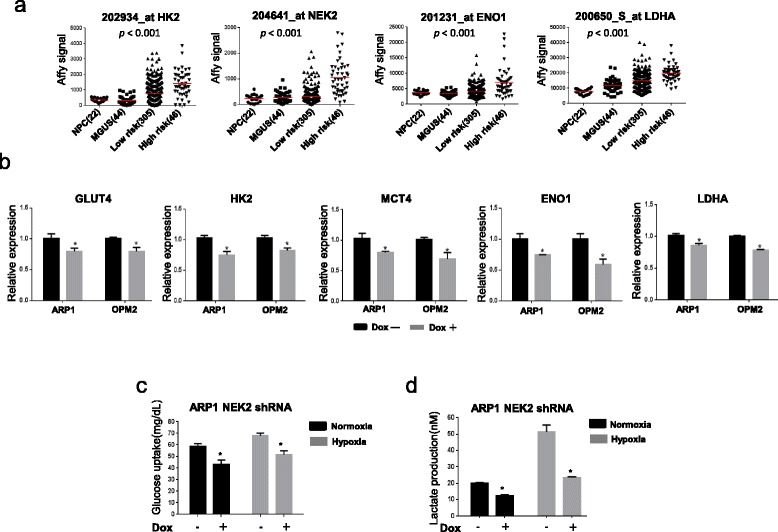
NEK2 regulates aerobic glycolysis in myeloma cells. **a** GEP analysis of NEK2, HK2, ENO1, and LDHA on plasma cells derived from normal healthy donors (*n* = 22), MGUS patients (*n* = 44), low-, and high- (*n* = 305) risk MM patients (*n* = 46). **b** Real-time PCR was performed to test the expression of GLUT4, NEK2, HK2, ENO1, and LDHA in NEK2 silenced ARP1 and OPM2 MM cell lines, *p < 0.001. **c**, **d** Glucose uptake and lactate production were analyzed in NEK2 knocked down ARP1 MM cells cultured at normoxia (*black column*) or hypoxia (*grey column*), *p < 0.05

PKM2 plays an important role in aerobic glycolysis. We then tested whether NEK2 alters aerobic glycolysis via regulating PKM2 expression. The expression of NEK2 and aerobic glycolysis genes was examined in plasma cells derived from 22 healthy subjects, 44 monoclonal gammopathy of undetermined significance (MGUS) patients, 305 low- and 46 high-risk myeloma patients using gene expression profiling (GEP). The expression of NEK2 and glycolysis-enhancing genes, such as hexokinase 2 (HK2), alpha-enolase (ENO1), and lactate dehydrogenase A (LDHA), was significantly increased in high-risk myeloma samples and positively correlated each other (Fig. 3a). We then confirmed these gene expressions in NEK2 silenced ARP1 and OPM2 myeloma cells by real-time PCR (Fig. 3b). Consistently, the expression of HK2, ENO1, LDHA, glucose transporter type 4 (Glut4), and monocarboxylate transporter 4 (MCT4) was downregulated in NEK2 silenced myeloma cells. To determine whether NEK2 regulates aerobic glycolysis, we tested glucose uptake and lactate production in NEK2 knockdown cells and control cells at normoxia or hypoxia (1% oxygen) conditions. As shown in Fig. 3c, d, both glucose uptake and lactate production decreased in NEK2 knockdown ARP1 and OPM2 myeloma cells compared to the control cells in both conditions.

### c-Myc transcriptionally regulates NEK2 expression

**Fig. 4:**
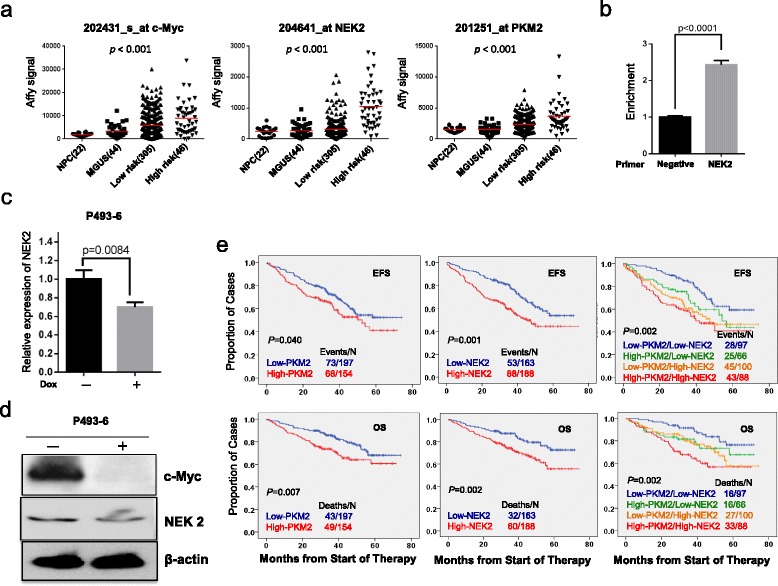
NEK2 is a transcriptional target of c-Myc. **a** *Dot-plots* present the expression of NEK2, c-Myc, and PKM2 in plasma cells of GEP derived from normal donors (*n* = 22), MGUS patients (*n* = 44), low- (*n* = 305), and high- (*n* = 46) risk MM patients (*p* < 0.001). **b** ChIP PCR detected binding of c-Myc to the promoter of Nek2 in P493-6 cells. IgG antibodies were used as negative control. **c** Real-time PCR shows the expression of NEK2 in P493-6 cells after silencing c-Myc. **d** Western blots show protein expression of NEK2, c-Myc, and β-actin in P493-6 cells with knocking down c-Myc. **e** Kaplan-Meier analyses of event-free survival (*top panels*) and overall survival (*bottom panels*) among MM patients with different expression levels of NEK2 and PKM2

Although NEK2 expression is increased in various cancers, the regulation of its expression remains unclear. It is known that NEK2 is a potential target of c-Myc from chromatin immunoprecipitation (ChIP) sequencing [49], and c-Myc regulates pyruvate kinase mRNA splicing in cancer by upregulation of hnRNPA1/2 and PBT [39]. Given that both NEK2 and c-Myc are involved in hnRNPA1/2 mediated-PKM splicing, we hypothesize that c-Myc induces PKM splicing may depend on upregulation of NEK2, at least partially. We compared the expression of NEK2, c-Myc and PKM2 in plasma cells derived from healthy donors, MGUS patients, low- and high-risk MM patients described above. As shown in Fig. 4a, the expression of c-Myc, NEK2, and PKM2 is positively correlated and increased significantly in high-risk MM samples (*p* < 0.001 by one-way ANOVA). A chromatin immunoprecipitation-qPCR (ChIP-qPCR) assay was performed using anti-c-Myc antibodies to pull down binding DNA in a human B cell line P493-6 that is stably transfected with EREB2-5 and the construct c-Myc-tet [50]. Consistently, NEK2 promoter DNA sequences were significantly enriched by c-Myc antibody pulling down in P493-6 cells without addition of doxycycline compared with addition of doxycycline (Fig. 4b). Addition doxycycline in P493-6 cells decreases c-Myc expression (Fig. 4d). NEK2 expression was significantly decreased at both transcription and protein levels following inhibition of c-Myc (Fig. 4c, d) further suggesting that c-Myc regulates the expression of NEK2.

To determine the clinical relevance of aerobic glycolysis signaling, Kaplan-Meier analyses of event-frees (EFS) and overall survivals (OS) were performed on 351 newly diagnosed myeloma patients. Myeloma patients with high PKM2 expression had shortened EFS (*p* = 0.040) and OS (*p* = 0.007), which is similar to NEK2 (EFS *p* = 0.001; OS *p* = 0.002) (Fig. 4e). To further investigate whether NEK2 has a synergistic or additive effects in patient outcome, the 351 myeloma patients were classified into 4 groups including low-NEK2/low-PKM2, low-NEK2/high-PKM2, low-PKM2/high-NEK2, and high-NEK2/high-PKM2; and Kaplan-Meier analyses showed clearly that the high NEK2/PKM2 group had the worst outcome in both EFS and OS (Fig. 4e).

### NEK2 contributes to c-Myc regulated aerobic glycolysis and cell proliferation

**Fig. 5:**
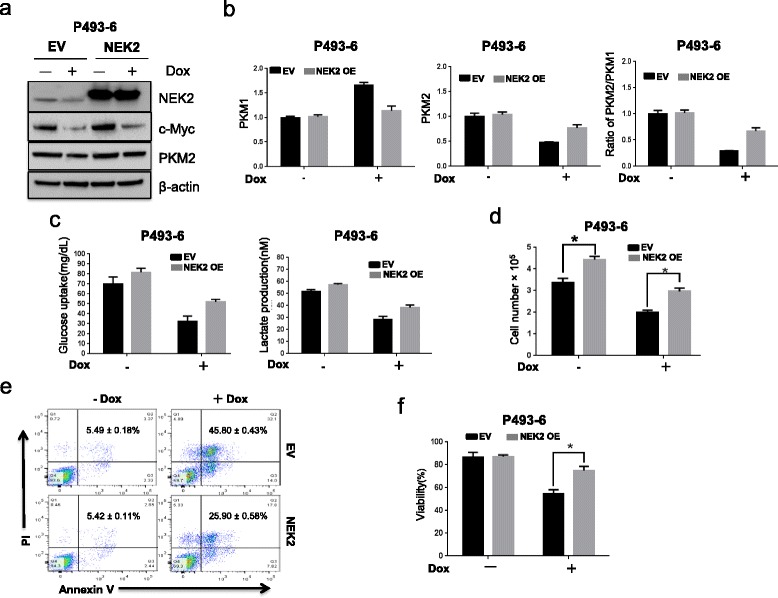
NEK2 mediates c-Myc-regulated aerobic glycolysis. **a** P493-6 cells with or without NEK2-OE were treated with Dox to inhibit c-Myc expression. Western blots show the protein expression of c-Myc, NEK2, and PKM2. **b** Real-time PCR shows the relative expression of PKM1 and PKM2 in P493-6 cells with altered expression of c-Myc and NEK2. **c** Glucose uptake and lactate production were evaluated in P493-6 cells with altered expression of c-Myc and NEK2. **d** Cell growth was analyzed in P493-6 cells with altered expression of c-Myc and NEK2 by trypan blue staining (*p < 0.05). **e** Flow cytometry analysis of apoptosis in P393-6 cells with silencing of c-Myc in the presense or absence of NEK2 overexpression using FITC-conjugated annexinV/PI staining. Apoptotic cells were annexinV positive. Representative pictures of FCM were shown with quantification of percentage of cells with apoptosis. Results from 3 independent experiments were shown. **f** Cell viability was analyzed in P493-6 cells with altered expression of c-Myc and NEK2 using trypan blue staining, *p < 0.05

hnRNPA1/2 were found to play an important role in c-Myc regulated aerobic glycolysis. Similar to hnRNPA1/2, the expression of NEK2 was regulated by c-Myc at transcription level in myeloma cells. We hypothesize that NEK2 plays a role in c-Myc-mediated aerobic glycolysis. To determine whether NEK2 is involved in c-Myc-mediated aerobic glycolysis, we examined the alternative splicing of PKM and aerobic glycolysis in NEK2 overexpressed P493-6. In P493-6 cells c-Myc expression was inhibited by addition of doxycycline. As shown in Fig. 5a, c-Myc was significantly downregulated upon addition of doxycycline leading to decrease PKM2 expression. However, the decreased expression of PKM2 was rescued in P493-6 cells overexpressed NEK2 and the ratio of PKM2/PKM1 decreased more than two folds in P493-6 cells with low c-Myc expression, while the expression ratio of PKM2/PKM1 changed slightly in P493-6 cells silenced c-Myc and overexpressed NEK2 (Fig. 5b). Consistently, with the expression alteration of PKM2 and PKM1, both glucose uptake and lactate production were significantly decreased in P493-6 cells with low c-Myc expression. However, P493-6 cells overexpressed NEK2 showed high glucose uptake and lactate production regardless of c-Myc alteration (Fig. 5c). These results indicate that NEK2 can partially neutralize downregulation of c-Myc-mediated decrease of the PKM2/PKM1 ratio and aerobic glycolysis. To further determine the functional role of NEK2 in c-Myc regulated aerobic glycolysis, we evaluated cell proliferation and cell viability in NEK2 OE and EV P493-6 cells after silencing of c-Myc. Notably, NEK2 OE P493-6 cells grow faster than EV cells in the presence or absence of c-Myc (Fig. 5d). Silence of c-Myc induced significantly P493-6 cell apoptosis (EV NEK2-OE = result in 45.80 ± 0.43%, 25.90 ± 0.58%; *p* < 0.05) (Fig. 5e). Moreover, P493-6 cells overexpressing NEK2 cells showed higher viability than those control cells (Fig. 5f). These results demonstrated that NEK2 is involved in c-Myc-regulated aerobic glycolysis which promotes cancer cell proliferation.

## Discussion

Almost one century ago, Dr. Warburg observed that cancer cells, unlike normal cells, rely on glycolysis to generate the energy needed for cellular processes rather than mitochondrial respiration despite of oxygen available [5]. Recently, some factors have been found to regulate Warburg effect including tumor microenvironment, stabilization of hypoxia inducible factor 1 (HIF1), oncogenic activation and/or tumor suppressor genes’ inhibition, mitochondrial dysfunction, glutamine metabolism, and post-translational modifications [2]. Our data from this study indicate that NEK2 plays an important role via regulating aerobic glycolysis resulting in MM cell proliferation. Reprogramming of energy metabolism is one of the eight hallmarks acquired during the multistep development of human tumors [51, 52]. Genomic instability, which causes genetic diversity, underlies these hallmarks [51, 52]. We have demonstrated that high NEK2 expression induces chromosomal instability and cancer cell proliferation [41]. In this study, we have shown that NEK2 binds and interacts with hnRNPA1 and hnRNPA2, which control pyruvate kinase mRNA splicing in cancer cells, and increases PKM2 expression and PKM2/PKM1 ratio in myeloma cells. The complex of hnRNPA1, hnRNPA2, and PTB binds to intronic sequences flanking exon 9 (contained in PKM1) and suppresses its splicing and activates exon 10 splicing of PKM (contained in PKM2), resulting in upregulation of PKM2 expression and downregulation of PKM1 [38]. Our RNA immunoprecipitation showed that NEK2 binds to intronic sequences flanking exon 9 of PKM pre-mRNA. Overexpression of NEK2 upregulates the expression of PMK2 while decreases PKM1 expression leading to increased PKM2/PKM1 ratio compared to control cells. Our data demonstrate that knockdown of NEK2 in myeloma cells decreased expression of PKM2 and the ratio of PKM2/PKM1. Knockdown of NEK2 also altered expression of critical genes involved in glycolysis under normoxia and/or hypoxia. The glucose uptake and lactate production were also impaired when NEK2 was knocked down. Because PKM2 is an essential enzyme for regulation of aerobic glycolysis in cancer cells, we further determine that NEK2 expression is increased in high-risk patients and positively correlates with aerobic glycolysis genes including HK2, ENO1, and LDHA. The subsequent assays show both glucose uptake and lactate production decrease in NEK2 silenced myeloma cells. The clinical data for survival analyses indicate that myeloma patients with high NEK2 and PKM2 had the shortened survival. Together, NEK2 promotes aerobic glycolysis through activating pyruvate kinase mRNA splicing in myeloma cells. NEK2 is a lethal target of c-Myc [49], we defined that c-Myc directly binds to the NEK2 promoter sequence and regulate its expression. We and others showed that c-Myc induced neoplastic tumor cells undergo high aerobic glycolysis in accordance with Warburg effect [53]. This effect demonstrates that most cancer cells take up large amount of glucose and convert them into lactic acid for generation of energy in the presence of oxygen but reduce rate of pyruvate oxidation. c-Myc is one of the most frequently deregulated oncogenes in human malignancies especially B cell lymphomas and multiple myeloma [54–56]. c-Myc increases PKM2 expression which regulates chromosome segregation and cell cycle G1/S transition as well as aerobic glycolysis in tumor cells [39]. In light with these studies, our ChIP-PCR confirmed that c-Myc directly binds to the promoter of NEK2 in c-Myc overexpressing P493-6 cells. Inhibition of c-Myc in P493-6 cells decreases the expression of NEK2 and PKM2. Given that NEK2 regulates PKM2 expression and that the expression and activity of NEK2 and PKM2 are controlled by c-Myc, NEK2 might be involved in c-Myc regulated aerobic glycolysis. This was supported by evidence that the PKM2/PKM1 ratio and aerobic glycolysis were significantly decreased in P493-6 cells by knocked down c-Myc, while overexpression of NEK2 blocked this decrease. Furthermore, knockdown of c-Myc-induced cell death and cell growth arrest can be rescued by overexpression of NEK2. We conclude that NEK2 is a novel c-Myc target for regulation of PKM splicing and aerobic glycolysis in myeloma. In general, our data shows the first evidence that NEK2 promotes aerobic glycolysis and provides mechanistic insights into how NEK2 regulates aerobic glycolysis in MM. Our study not only uncovers a new function of NEK2 but also contributes to study aerobic glycolysis mechanism in cancer. Previous studies have demonstrated that NEK2 promotes drug resistance in multiple myeloma [41], it is very likely that enhanced aerobic glycolysis by NEK2 may contribute to its function in drug resistance. We also speculate that targeting aerobic glycolysis may overcome NEK2 induced drug resistance in multiple myeloma.

## Conclusions

**Fig. 6:**
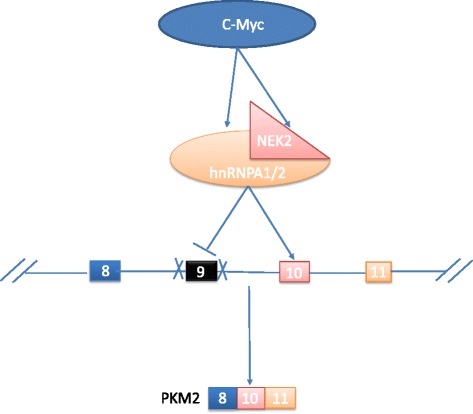
Schematic model of NEK2-mediated aerobic glycolysis through splicing of PKM1/2. c-Myc enhances the transcription of NEK2 and hnRNPA1/2, then NEK2 and hnRNPA1/2 complex bind to the intronic sequences flanking exon 9 of PKM pre-mRNA to out splicing exon 10 result in elevated expression of PKM2 and increased aerobic glycolysis

In this study, we characterize NEK2 as a new positive regulator of aerobic glycolysis through regulating PKM pre-mRNA splicing. NEK2 is a direct target of the transcription factor c-Myc and is involved in c-Myc-induced aerobic glycolysis. We demonstrate that NEK2 may interact with hnRNPA1 and hnRNPA2 proteins to regulate PKM splicing and aerobic glycolysis (Fig. 6).

## Abbreviations

ChIP-qPCR: Chromatin immunoprecipitation-QPCR
Co-IP: Co-immunoprecipitation
Dox: Doxycycline
EFS: Kaplan-Meier analyses of event-frees
GEP: Gene expression profile
OS: Overall survivals
RIP: RNA immunoprecipitation
shRNA: Small hairpin RNA
TAP-MS: Tandem affinity purification plus mass spectrometry
